# Scent of a father: Paternal body odors boost interbrain synchrony

**DOI:** 10.1126/sciadv.aed6110

**Published:** 2026-07-15

**Authors:** Yaara Endevelt-Shapira, Linoy Schwartz, Ruth Feldman

**Affiliations:** ^1^ Center for Developmental Social Neuroscience, Reichman University, Herzliya, Israel.; ^2^ Institute for Learning & Brain Sciences, University of Washington, Seattle, WA, United States.

## Abstract

Olfactory cues are ancient signals and mammalian young utilize maternal body odors (BO) to form a bond to their habitat in the absence of the mother, strengthen associative learning, and grow a social brain. Whether infants respond to their father’s BO and how paternal BO impact their brain maturation is still unknown. Utilizing ecologically-valid paradigms with dual-electroencephalography recordings, we measured infant-father and infant-stranger interbrain synchrony during naturalistic interactions and assessed the effects of paternal BO. Infants show greater interbrain synchrony with father compared to a stranger male. Paternal BO increase infant-stranger interbrain synchrony and enhance the father-typical positive arousal, providing evidence that infants process their father’s odor and can invoke his presence in his absence. Fathers contribute to brain maturation by enhancing cross-hemisphere connectivity in alpha rhythm that sustains attention regulation during stimulating social interactions. Paternal olfactory cues can invoke infants’ associative learning and may push the development of more complex neural and behavioral competencies.

## Teaser

Infants process their father’s body odor and use it to enhance interbrain synchrony and recreate father’s presence in his absence.

## INTRODUCTION

The evolution of mammals placed the biology of maternal care at the forefront, and decades of neuroscientific research described the role of maternal social cues in shaping the infant’s brain maturation and sociality. The neurobiology of fatherhood, on the other hand, has only recently become an important topic, alongside the growing involvement of men in infant care and family life (*1*). Studies in humans and bi-parental mammals have shown that a father’s brain undergoes substantial reorganization upon the birth of his infant, accompanied by hormonal fluctuations and increased neural plasticity that parallel those of a mother’s (*2*, *3*). Still, changes in a father’s brain and neurobiology are much more variable across individuals and cultures compared to mother’s, necessitate active involvement in childcare, and the impact of fathering on the child depends on the degree of exposure to paternal stimuli. Moreover, neural changes in fathers implicate more complex processes and recruit higher-order cortical areas (*4*), as compared to the more ancient networks that underpin maternal care. Studies have shown that fathers’ social signals and sensory cues push infants toward greater exploration and mastery (*5*–*8*), elicit higher positive arousal, and stimulate the acquisition of attentive and cognitive skills (*9*–*11*), whereas maternal sensory cues promote safety, familiarity, and the downregulation of arousal (*7*, *12*).

Olfactory cues are ancient signals that stamp mother and habitat with the signature of the bond; olfactory cues are the only sensory signals a mother can leave behind to retain her presence in her absence. Olfactory learning begins in utero and human newborns are selectively responsive to the odor of their own amniotic fluid and can detect and orient toward lactating-breast odors (*13*, *14*). Increasing familiarity then continues through early caregiving experiences, such as feeding, carrying, and cuddling, that cement the role of maternal chemosignals in infant socialization. Maternal body odors reduce pain in newborns (*15*), increase infant attention to faces and eyes (*16*), shape face categorization (*17*), and attenuate neural response to fearful faces (*18*). Maternal body odors play a vital role in facilitating social communication and orienting infants toward species-specific social cues, which in humans involve orientation to faces and eyes (*16*–*19*).

In contrast to maternal chemosignals, the role of fathers’ olfactory cues is unknown. No study to date has tested whether infants process and respond to their father’s body odors, and what are the neural correlates of the paternal odor effects. Do paternal chemosignals impact infant development by increasing the child’s sense of safety, attention to faces, and approach behavior, similar to the mother’s olfactory signals, or they ride on different, complementary mechanisms that sustain paternal care, such as bursts of arousal, exploratory focus, and heightened engagement typical of the father?

While there have been no empirical studies on infants’ detection of paternal body odors, anthropological field studies describe a ceremony by which a newborn is introduced to his father through his smell in the Wik Monkan tribe. During the ceremony “When he had received the child in his arms, the father passed his hand under his axilla and rubbed the sweat, or ‘smell’ as the natives say, on the head of the child” (*20*). Traditional wisdom appreciates the role of paternal body odors in consolidating the father-infant bond and underscores the need to perform an active public event by which a child learns to detect his own father’s odors from those of other men in the community. This is important, as the child meets the father’s odors only upon birth and not in-utero and paternal olfactory cues may be at a disadvantage in their salience compared to the mother’s odors. Such ceremony highlights the importance of initiating father involvement in childcare through concrete sensory experiences and calls to employ ecologically-valid paradigms when studying the uniqueness of the father’s body odors. Consistent with this tradition, the current study utilizes a set of ecological paradigms to examine the effects of the father’s olfactory cues on the infant’s brain and behavior.

One important mechanism of infant brain maturation that has received increasing attention in human neuroscience is interbrain synchrony. Hyperscanning studies, in which the brains of two or more individuals are measured simultaneously during a social act to assess the synchrony between their activations and rhythms, offer an ecologically-valid method for studying the social brain “in the wild” and test how sensory cues impact the connection between brains (*21*).

Hyperscanning studies of mothers and infants have shown that mothers synchronize their brain with the infant’s social brain, particularly with temporal regions that undergo substantial maturation in the second six months of life. Utilizing interbrain synchrony mechanisms, mothers can provide critical sensory inputs during a sensitive period for social brain maturation that tune the child’s brain to the social ecology and its distinctive features. When mothers engage in the human-specific social repertoire—orientation to face, shared gaze, expression of positive affect, and affectionate touch—the degree of mother-infant interbrain synchrony increases. In contrast, when infants avert their gaze and mothers engage in intrusive, uncoordinated behavior, the coupling between their brain is decreased (*22*, *23*). Studies further showed that interbrain synchrony encodes the signature of the bond, and infants create tighter neural synchrony with their mothers as compared to a strange female (*24*).

Father-infant interbrain synchrony is still relatively unexplored. The one existing study (*25*) showed that infants form greater neural synchrony with their fathers as compared to a stranger, indicating that interbrain processes with the father similarly retain the signature of the bond. This study further indicated that stranger-infant neural synchrony is sensitive to sensory signals and increases in the presence of infant-directed speech.

Two hyperscanning studies of fathers and older children (*26*, *27*) provided further validation that children form tighter interbrain synchrony with their fathers compared to strangers, and that neural coupling increases when the interaction involves collaboration and coordinated behavior. Consistent with the human and animal studies on the neurobiology of fatherhood (*28*, *29*), when fathers reported greater involvement in childcare, the degree of interbrain synchrony increased, suggesting that father-child neural coupling depends on the amount of paternal sensory stimuli the child receives.

Interbrain mechanisms are sensitive to maternal olfactory cues. Utilizing an ecological paradigm of infants’ interactions with their mother and an unfamiliar mother from the same community, we showed that infants synchronize their brain more with mother than with a stranger, but the presence of maternal body odors eliminated this difference (*24*). These findings echo the ancient practice of allomothering observed across all primate species and traditional human societies, where women of the social group are involved in the caring for babies in the absence of the mother (*30*), who leaves her olfactory cues in the social environment. The mother’s presence experienced only through her body odors increases infant visual attention to social signals, augments its positive engagement, and improves safety when interacting with a stranger, enhancing the interbrain synchrony her child forms with the stranger (*24*).

What about the father’s body odors? Do they also serve as signals that can retain the father’s presence in his absence and increase infant neural synchrony with unfamiliar men in the community, expanding the infant’s trust and opening the road from the intimacy of the family to the larger social group? Employing the same ecological paradigm, the current study investigates this question, targeting two unexplored topics in the neurobiology of fatherhood; the role of father’s olfactory cues and the mechanisms of interbrain synchrony between fathers and infants.

**Fig. 1.**
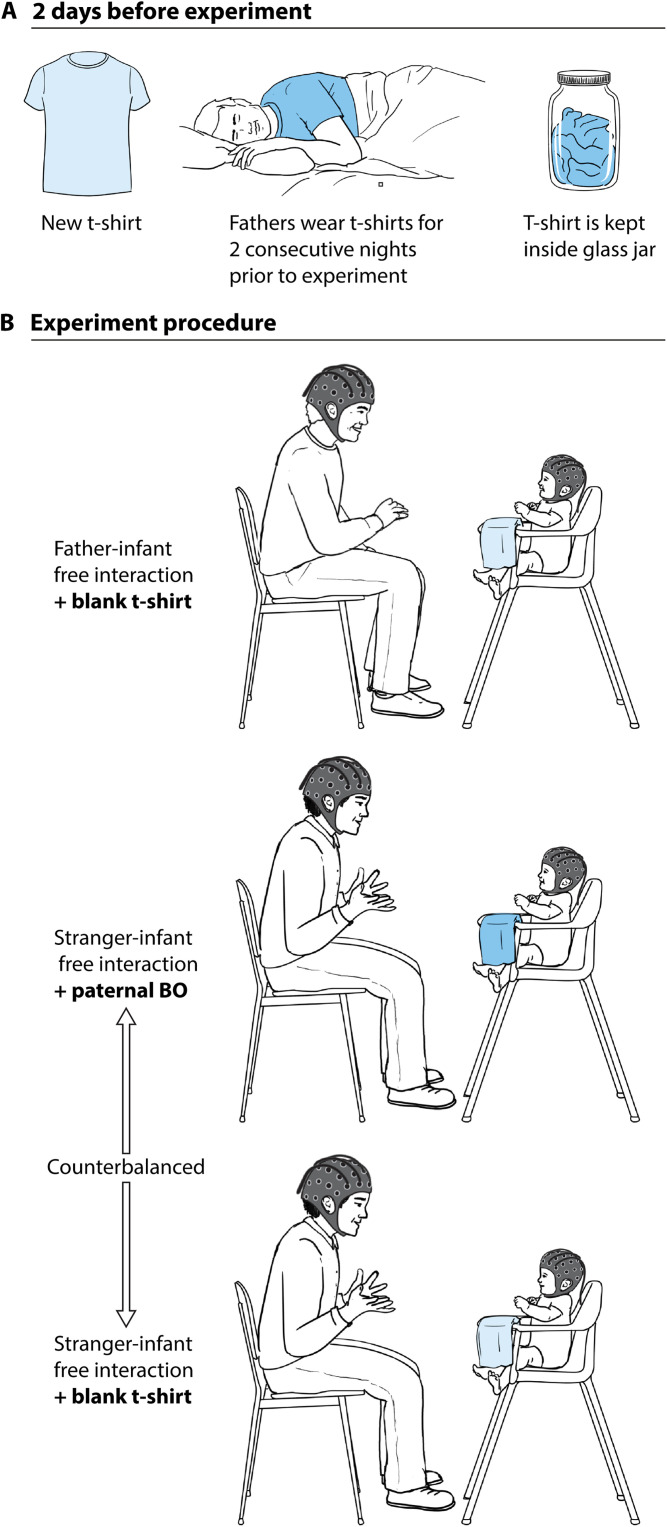
Experimental procedures. (**A**) BO collection. Two days before the experiment, fathers were given 100% cotton T-shirts to wear for two consecutive nights before the experiment. Between nights, the T-shirts were kept inside a closed glass jar and stored in the participants’ freezer. (**B**) Infant-adult paradigms. Infant, father and the stranger were fitted with EEG electrodes and participated in face-to-face free interaction paradigms. One infant-father interaction and two consecutive infant-stranger interactions were conducted. For the infant-stranger interactions, one was conducted with continuous exposure to paternal BO and the other in the presence of a new T-shirt placed in the same position. The two odor conditions were counterbalanced. Illustration credit: M. Harel.

We tested four hypotheses related to core processes that underpin infant-father interbrain synchrony (Fig. 1, A and B). Specifically, in the current study we examined infant-adult concurrent, nondirectional neural synchrony. First, we expected greater interbrain synchrony between infants and their own fathers as compared to an unfamiliar father from the same community. Second, we expected that exposure to paternal body odors would retain the father’s presence in his absence and increase neural synchrony between the infant and an unfamiliar male. Third, consistent with the finding for mothers, we hypothesized that infants’ social behavior would be influenced by their father’s body odors, and that changes in behavioral measures would be associated with the effects of the father’s body odor on interbrain synchrony. This was tested as an exploratory hypothesis using the same behavioral measures as those we tested in mothers; infants’ social attention, positive arousal, and safety-approach behavior. Given that paternal caregiving has been shown to be more stimulating than maternal caregiving, we expected infants’ positive arousal to increase in response to the father’s body odor. Moreover, consistent with findings on the consolidation of the father’s brain (*28*, *29*), we conducted an exploratory analysis to examine whether greater father involvement in childcare would be associated with stronger effects of paternal body odor on infant-stranger interbrain synchrony.

Our final hypothesis considered potential differences in the cognitive and neural processes associated with fatherhood compared to those previously reported in mothers (*4*, *31*). As compared to the theta rhythm that sustain infant-mother interbrain synchrony, we explored whether alpha rhythm provides the venue for interbrain coupling with fathers. The alpha rhythm develops in the second half of the first year and is associated with the structural and functional maturation of cortical networks during that period (*32*, *33*). Alpha oscillations support infant attention (*34*), motion observation and execution (*35*), working memory, and inhibitory control (*36*) and we thus explored whether father-child interbrain synchrony would ride on the alpha rhythm.

## RESULTS

### Higher father-infant inter brain synchrony compared to stranger-infant

To test our first hypothesis, we used weighted phase lag index (wPLI) connectivity scores obtained from 40 infants who completed both father-infant and stranger-infant interactions. Based on our previous findings (*24*), we chose to focus on interbrain synchrony between infants’ temporal regions and adults’ central and temporal regions. Our primary analysis used a conservative approach, applying nonparametric Wilcoxon signed-rank tests to all eight possible infant-adult connectivity combinations. All results were Bonferroni-corrected to account for the eight comparisons. Specifically, each *P* value was divided by the number of comparisons and compared against a standard alpha level of 0.05 to determine significance.

**Fig. 2.**
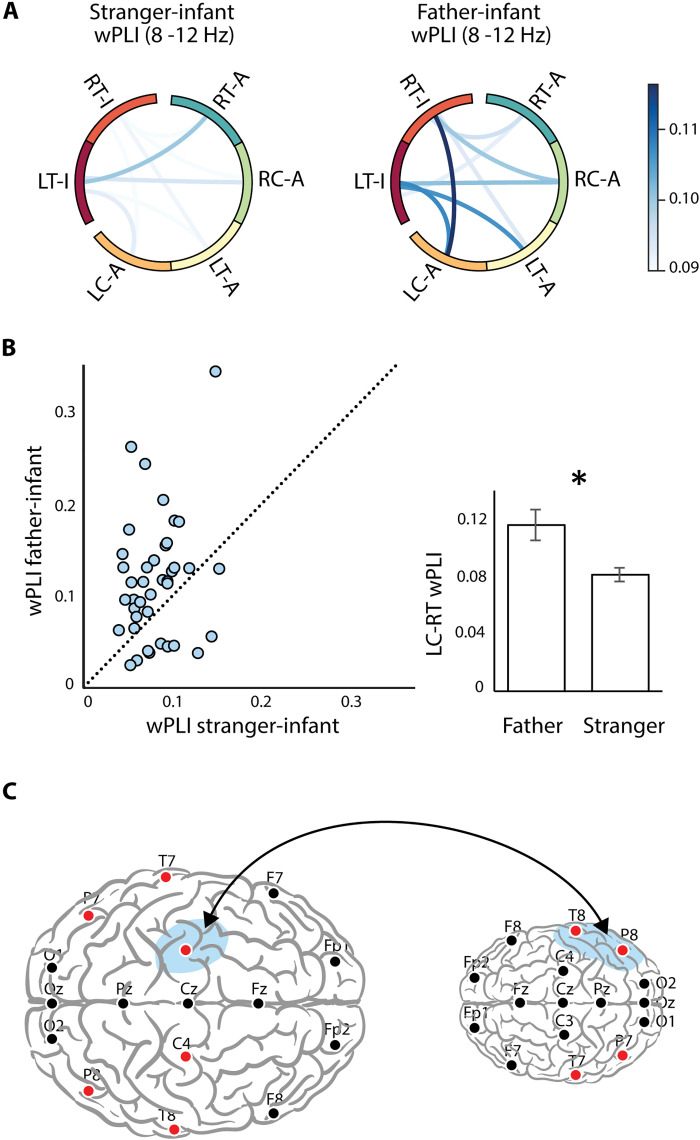
Higher interbrain neural synchrony during father-infant compared with stranger-infant interaction. (**A**) Visualization of connectivity values (wPLI) during strager-infant (right) and father-infant (right) face-to-face conditions. Each circle represents mean connectivity values for 8 combinations (4 regions for adult × 2 regions for infants). Within each circle, interbrain connections between infants’ regions (indicated by the suffix “I”) and adults’ regions (indicated by the suffix “A”) are shown. Darker shades represent greater values of inter-brain connectivity (wPLI scores). Abbreviations: Left Central (LC); Left Temporal (LT); Right Central RC); Right Temporal (RT). (**B**) Interbrain neural synchrony between the left central area of the adult and the right temporal area of the infant. Each circle represents the connectivity score of a single participant following father-infant (*y* axis) and stranger-infant (*x* axis). The diagonal line reflects the unit slope line (*x* = *y*) such that if points accumulate above the line, then values are greater for father-infant, and if they accumulate under the line, then values are greater for stranger-infant. The bar graph represents the quantified results of the data shown in the scatter plot (N = 40, Z = 2.77, W = 616, *P* corrected = 0.04). (**C**) Illustration of infant-adult interbrain neural synchrony, between adult’s left-central and infant’s right temporal.

While no significant connections were found in theta frequency band (all *P* > 0.1 before correction for multiple comparisons), the analysis in alpha frequency band (8–12 Hz) revealed a significant connection between the adult left central and infants’ right temporal regions, indicating higher father-infant interbrain connectivity compared to the stranger-infant interaction. (N = 40, Father-infant: 0.116 ± 0.067, Stranger-infant: 0.081 ± 0.029, Z = 2.77, W = 616, *P* = 0.005, *P* corrected = 0.04, Rank-Biserial effect size = 0.50, 95% CI [0.19, 0.72]) (Fig. 2, A, B and C). [See fig. S1A for the same analysis with phase locking values (PLV) (*37*) and fig. S2A for the same analysis in low and high alpha frequency bands.]

To further verify that the observed inter-brain synchrony is differentiated from spurious synchrony that could be driven by common intrinsic properties of the signal or consistent external perturbation during the experiment, we randomly shuffled the epochs of one member in each dyad 1000 times and compared the original connectivity values with the mean connectivity values obtained from the shuffled data. The analysis revealed a significant difference between the original and the shuffle scores of the RC-RT connection (N = 40, original Father-infant: 0.116 ± 0.067, Shuffle data: 0.086 ± 0.014, Z = 2.58, W = 218, *P* = 0.009, Rank-Biserial effect size = 0.47, 95% CI [0.15, 0.70]).

### Higher inter-brain synchrony during exposure to paternal BO compared no blank odor

To test our second hypothesis, we used the wPLI connectivity scores obtained from 40 infants who completed the interactions with the stranger in both odor conditions (counterbalanced).

**Fig. 3.**
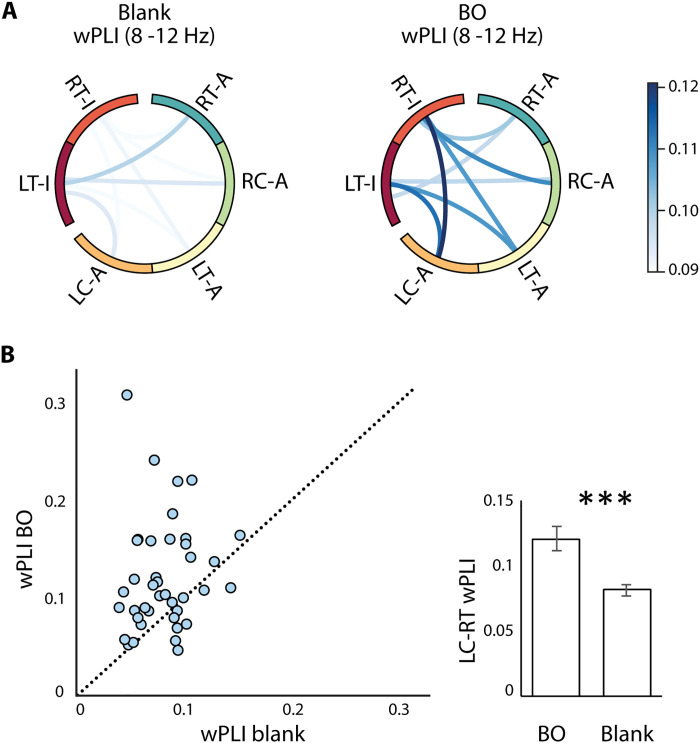
Increased interbrain neural synchrony following exposure to paternal body odor. (**A**) Visualization of connectivity values (wPLI) in the blank (left) and the BO (right) conditions. Each circle represents mean connectivity values for 8 combinations. Within each circle, interbrain connections between infants’ regions (indicated by the suffix “I”) and adults’ regions (indicated by the suffix “A”) are shown. Darker shades represent greater values of inter-brain connectivity (wPLI scores). (**B**) Interbrain neural synchrony between the left central area of the adult and the right temporal area of the infant. Each circle represents the connectivity score of a single participant following BO (*y* axis) and blank (*x* axis). The diagonal line reflects the unit slope line (*x* = *y*) such that if points accumulate above the line, then values are greater for BO, and if they accumulate under the line, then values are greater for blank. The bar graph represents the quantified results of the data shown in the scatter plot (N = 40, Z = 3.87, W = 698, *P* = 0.0005).

While no significant connections were found in theta frequency band (all *P* > 0.19), the analysis in alpha frequency band (8–12 Hz) revealed significantly higher connectivity in BO condition relative to blank between the left central area of the stranger and the right temporal area of the infant (N = 40, BO: 0.121 ± 0.057, blank: 0.081 ± 0.027, Z = 3.87, W = 698, *P* = 0.00004, *P* corrected = 0.0005, Rank-Biserial effect size = 0.70, 95% CI [0.47, 0.84]) (Fig. 3, A and B). (See fig. S1B for the same analysis with PLV and fig. S2B for the same analysis in low and high alpha frequency bands.) To further explore the effects of paternal BO on neural connectivity between infants and adults, we also compared directly the connectivity values between the BO condition and the father-infant interaction, and found no significant differences for all connectivity combinations (all *P* > 0.13, before correction for multiple comparisons). Specifically, we compared the neural synchrony scores of the left central area of the adult and the right temporal area of the infant between the father-infant and BO conditions. This comparison revealed no difference between father and BO conditions in neural synchrony scores (Z = 0.44, W = 377, *P* = 0.67) (see fig. S3).

### Increased infants’ positive arousal following exposure to paternal BO

To test our third exploratory hypothesis, we examined the effects of paternal BO on infant social behavior: visual attention, safety and engagement, and positive arousal. For this purpose, each paradigm was coded offline using two well-validated coding schemes: the social behavior global coding scheme Coding Interactive Behavior (CIB) and microlevel second-by-second coding scheme.

### Infant visual attention

**Fig. 4.**
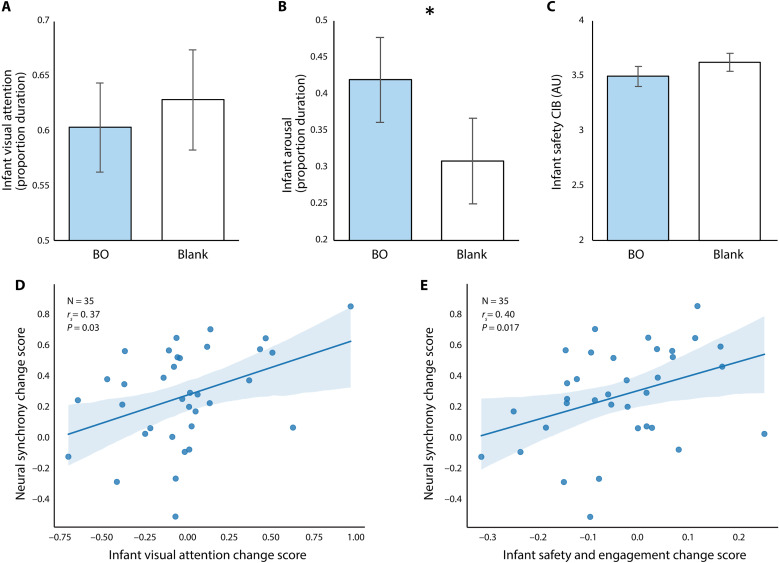
Increased positive arousal following exposure to paternal BO. Means ± SEM of (**A**) infants’ visual attention proportion duration, (**B**) infants’ positive arousal proportion duration, and (**C**) infants’ safety and engagement scores obtained in two odor conditions: blank (white) and BO (light blue). (**D**) The relation between neural synchrony change score of each dyad and the infant’s gaze change score (N = 35, *r_s_* = 0.37, *P* = 0.03) and (**E**) The relation between neural synchrony change score of each dyad and the infant’s safety and engagement change score (N = 35, *r_s_* = 0.40, *P* = 0.017).

First, we explored Infant social gaze, which is a key nonverbal social behavior, that was previously shown to mediate the elevation in interbrain synchrony following exposure to maternal BO (*24*). While no difference emerged between conditions, (BO: 0.60 ± 0.24, Blank: 0.63 ± 0.27, Z = −1.3, *W* = 236, *P* = 0.2, Fig. 4A), interbrain neural synchrony change score correlated with the visual attention change score (*r_s_* = 0.37, *P* = 0.03, Fig. 4D), such that greater increase in visual attention following exposure to BO is associated with greater increase in neural synchrony.

### Positive arousal

Next, we tested the effects of paternal BO on infant’s positive arousal obtained from the microcoding. Infant social arousal was higher in the BO compared with blank condition (N = 35, BO: 0.42 ± 0.34, Blank: 0.31 ± 0.34, Z = 2.0, *W* = 372, *P* = 0.044, Rank-Biserial effect size = 0.41, 95% CI [0.037, 0.681], Fig. 4B). However, the degree of increase in positive arousal in the BO condition was not associated with the change in neural synchrony (*r_s_* = 0.02, *P* = 0.94).

### Safety and engagement

Using the CIB global scores, we analyzed effects of paternal BO on infants’ sense of safety, approach, and social engagement. Infant safety and engagement scores were not different between BO and blank conditions (BO = 3.50 ± 0.54, blank = 3.60 ± 0.49, Z = −1.51, *W* = 209, *P* = 0.13, Fig. 4C). However, the degree of increase in infant engagement in the BO condition was significantly associated with the change in neural synchrony (*r_s_* = 0.40, *P* = 0.017, Fig. 4E), such that greater increase in infant social engagement following exposure to BO is associated with greater increase in neural synchrony.

Finally, to examine whether paternal involvement was associated with the effect of paternal BO on interbrain synchrony, a Spearman correlation analysis was conducted between paternal involvement scores and change in neural synchrony. The results revealed a significant correlation between paternal involvement scores and interbrain synchrony change scores (N = 33, *r*_s_ = 0.38, *P* = 0.03) (see fig. S4).

## DISCUSSION

The current study focused on the neurobiology of fatherhood—a topic of increasing importance for men, women, and families—and addressed the unique mechanisms by which a father’s sensory cues impact his infant’s developing brain. We targeted two unexplored issues in the neuroscience of paternal caregiving. First, we examined whether infants are affected by their father’s body odor. Second, we investigated the processes that underpin infant-father concurrent, nondirectional interbrain synchrony. Integrating these two timely topics, our study uniquely described the ways by which a father’s presence is retained in his absence through his body odors and functions to boost the infant’s interbrain synchrony with unfamiliar males of the social group. Overall, our findings chart a two-brain perspective on how fathers tune their infants’ brain to social living by utilizing olfactory cues.

Our four hypotheses were confirmed and demonstrate both a basic similarity with our previous findings on maternal body odors alongside important differences. Like mothers, infants engaged in tighter interbrain synchrony with their own fathers as compared to an unfamiliar man. This suggests that by the second half year of life, the father-infant bond is already imprinted in the infant’s brain and comes to life every time father and child engage in social interactions. Second, we showed that the presence of the father’s body odors boosts the synchrony between the infant’s brain and that of an unfamiliar male from the community. Interbrain synchrony levels in the shuffled father-infant data were similar to the stranger-blank condition, indicating no true synchrony with a stranger, whereas paternal BO induced neural synchrony similar to the real father-infant condition. Our findings, therefore, provide evidence that infants are sensitive to their fathers’ olfactory cues. The findings suggest that fathers’ olfactory cues alone may be sufficient to elicit infant-stranger neural synchrony, even in the absence of other sensory cues. Infants can recreate the father’s presence from the odors he leaves behind and use these cues to increase their brain’s coordination with a stranger’s brain.

Third, we showed that processes of father-infant interbrain synchrony are different from those observed in inter-brain synchrony with mother. Neural synchrony with the father rides on the alpha rhythm, which emerges later in development as compared to the theta rhythm of the mother-infant neural synchrony that predominates in infancy. Father-infant interbrain synchrony also activates a cross-hemispheric connection between the left and right hemispheres of father and child, as compared to the right-to-right hemisphere connectivity with mother.

Olfactory cues are among the earliest sensory signals infants are exposed to before birth; hence, mothers have a significant advantage in the infant’s familiarity with their odor. Maternal chemosignals are evolutionary ancient. Terrestrial mammals rely on their sense of smell to perceive emotions and communicate socially, and maternal odor is the primary vehicle for the formation of the mother-infant bond in rodents (*38*). There is a growing evidence for meaningful social chemosignaling in humans as well (*19*, *39*–*41*). Human social chemosignals convey emotional states, such as happiness (*42*) and fear (*43*, *44*), and can influence aggression (*45*), mate selection (*46*), sexual arousal (*40*), nonromantic social bonding (*47*), and infant bonding (*19*, *48*, *49*). The findings that infants respond to their father’s odors show that paternal chemosignals are processed in the infant’s brain. Paternal olfactory cues may open the developing brain to a unique mode of two-brain communication the infant may later utilize in his social life. Our findings are consistent with positions indicating that the parental brain is characterized by a high degree of plasticity during the post-birth period (*50*) and there are multiple pathways by which the brain basis of attachment can consolidate (*51*, *52*). Our findings importantly show that infants’ sensitivity to parental olfactory cues are not limited to biological mothers, thereby not solely relying on pregnancy and nursing, and can be found in fathers. It is thus likely that the parental olfactory effect can be found in adoptive parents and involved grandparents, but such hypothesis should be specifically tested.

Our previous study with mothers that utilized the same paradigm highlighted a specific interbrain connection; between the mother’s right central region and the infant’s right temporal region in the theta frequency band (*24*). In the current study, the same link was observed, with two distinguishing features. First, while exact localization of neural regions is not possible with EEG and future research using other methods is required to identify the brain areas detected here with greater specificity, our results consistently highlight cross-hemispheric connectivity, between the father’s left central region and the infant’s right temporal region.

Fathers may recruit different mechanisms during interactions with their infants compared to mothers, which may provide developmental support to cognitive and attentive processes and to those associated with skill learning and tool use, while the infants themselves may rely on similar cognitive and neural processes when interacting with either parent, as the infant right temporal region is involved in interbrain connections with both mothers and fathers. The infants, in turn, may learn to use these cognitive, attentive, and motor execution processes during interactions with their father. While the right hemisphere is widely regarded to play a dominant role in emotion processing, the left sensorimotor cortex shows greater specialization for skilled movement and visuomotor processing and control (*53*–*55*). This is consistent with research on the father-child relationship indicating that father-child play contains skill-learning, explanations, and exploration of the environment (*5*, *10*, *12*). Despite the fact that interbrain synchrony in fathers was localized to the left central region and in mothers to the right central region, in both studies the parental regions showed connectivity with the infant’s right temporal regions. The right temporal cortex is a key area of the social brain, particularly the superior temporal sulcus (STS) implicated in simulation, mentalization, attention, and biological motion detection (*56*–*59*).

The second distinguishing feature was that the parent-central-infant-temporal connection in fathers utilized the alpha frequency band rather than the theta found in mothers. These findings were consistent across both low- and high-alpha frequency ranges (fig. S2). The alpha rhythm develops during the second half of the first year of life (*32*, *33*), and is the dominant frequency in the human scalp EEG of adults (*60*). Alpha rhythm over parieto-occipital regions is primarily modulated by visual inputs (*61*). The alpha rhythm is also associated with arousal and attention mechanisms and is not restricted to the visual domain; alpha-mediated attentional gating has been observed across a variety of attention tasks in multiple sensory modalities (*62*). In infants, alpha oscillations are associated with visual processing (*63*), attention (*34*), motion observation and execution (*35*), working memory and inhibitory control (*36*, *64*, *65*). The mu rhythm, which occurs in the same frequency band as the alpha, typically culminates over central regions that mark the human sensorimotor cortex (*66*). The sensorimotor cortex, which is the paternal brain region implicated in the brain-to-brain synchrony observed here is associated with observation of motor action (*66*). Moreover, mu rhythm is associated with social skills, including joint attention (*67*) processing of social context (*68*) and the social coordination of movements (*69*). Inter-brain synchrony has been observed in the alpha frequency range between pairs engaged in spontaneous reciprocal imitation (*70*). It appears that the synchrony infants create with their mother and father support different cognitive and neural processes. The theta right-hemispheric coupling during mother-infant interaction may function to support emotional processes, whereas the alpha cross-hemispheric coupling in fathers sustains more controlled attention regulatory processes.

The paternal olfactory effect, like the maternal, also influenced infants’ behavior. Father odors increased infant positive arousal during interaction with a strange male in the presence of the father’s body odors to a level even higher than that observed with the father. Behavioral studies of father-child interaction describe the father-typical style as containing frequent peaks of positive arousal, like high-energy laugh, positive vocalizations, or squeaks of joy accompanied by excited body movements. Fathers use this high positive arousal to orient children to the environment, encourage joint attention, and engage in mutual exploration, dubbing the father-child behavioral rhythms as the “rhythms of exploration” in comparison with the “rhythms of safety” of mothers (*5*–*8*). Our findings show that such father-typical highly-arousing play can be elicited in infants when the father’s presence is invoked merely by his body odors. It has been shown that human body odor carries an individual signature that enables olfactory kin recognition beginning in utero and continuing through the early experiences of feeding, carrying, and cuddling. Through these repeated exposures in the context of bonding, infants form associative learning, such as between the maternal body odor and the feelings of safety (*18*). Whether the effects of paternal body odor occur subliminally without conscious awareness or are consciously perceived by the infant, our findings suggest that infants may form similar associative learning with their fathers through his body odor. Our findings, therefore, open the possibility to a new understanding on the role of olfactory cues in bonding. Mothers may have more frequent opportunities for such learning, but our findings suggest that similar mechanisms are likely activated when infants perceive their fathers’ body odors and invoke the memory of their presence (*49*). While positive arousal showed an overall mean-level increase when infants interacted with a stranger in the presence of their father’s BO, and levels of positive arousal remained stable across the first and second minutes of the interaction (fig. S5), the increase in positive arousal was not coupled with the changes in neural coupling.

The more “mother-type” behaviors, such as looking at faces and safety-approach behavior that increased with maternal odors did not show an overall mean-level increase when infants interacted with a stranger in the presence of their father’s BO. Still, the more the infant looked at the stranger’s face and showed safety-engagement behavior, the greater was the boost in neural coupling with the stranger in the presence of the father’s odors.

These results suggest that two processes may occur following exposure to paternal body odor. First, there may be a general increase in interbrain synchrony and positive arousal, which are not necessarily coupled. Second, exposure to paternal body odor may specifically enhance the coupling between neural activity and specific behavioral responses. In order to understand the observed differences between maternal and paternal body odor effects on infant behavior and interbrain synchrony, future studies should examine exposure to both maternal and paternal body odor within the same experimental design. To further explore the neural and behavioral processes following exposure to body odor, future studies should be specifically designed to address this question. Odors should be delivered via an olfactometer in a controlled manner, with precisely defined durations and temporal jitter, allowing comparisons between moments when infants are exposed to odors and control periods within the same interaction.

While exposure to maternal body odor occurs early and naturally, exposure to paternal odors requires more active involvement and intentional proximity. The influence of paternal olfactory cues may thus vary according to the amount of time and the range of caregiving activities the father provides to his infant. Consistently, we found a significant correlation between paternal involvement in childcare and the impact of paternal BO on interbrain synchrony (see fig. S4). It is possible that additional factors modulate the effects of paternal body odor, including the infant’s broader ecology, as well as the quality of activities and interactions fathers engage in with their infants within the home environment. Moreover, the effects of paternal BO on interbrain synchrony may reflect individual differences in neurodevelopment of either child or father. Future work incorporating direct measures of neural maturation will be important to determine whether BO-related effects are associated with specific neurodevelopmental markers.

Future research in populations at elevated likelihood for developmental differences, such as infant siblings of children with autism spectrum disorder (ASD) may be particularly informative in this regard, as some prior work has suggested alterations in interbrain coupling during social interactions in ASD (*71*). Prior work has shown that imbuing objects with the mother’s body odor can significantly enhance imitation in children with ASD (*72*). This raises the possibility that exposure to socially relevant chemosignals may enhance interbrain synchrony in this population. Future research should examine how exposure to parental body odor influences social behavior and interbrain synchrony in infants at increased risk for ASD.

Limitations: Several study limitations should be acknowledged. First, it is important to note that caregiving behaviors vary significantly across individuals and are not strictly determined by biological sex. Any discussion on the contrast between father-infant and mother-infant interactions should be interpreted with caution and may reflect differences in caregiving profiles, personal styles, involvement in childcare, parental mental state, and other individual factors. Future studies with larger sample sizes that include both mothers and fathers within the same design will be essential to empirically examine individual variability in caregiving behaviors beyond biological sex alone, and clarify how these factors relate to interbrain synchrony and infant behavior.

Second, infants’ age range in the current study spanned a period of developmental change in alpha rhythm maturation. Although we did not observe a significant association between infant age and father-infant interbrain synchrony (see fig. S6), age-related heterogeneity remains a limitation of the study. Future research may focus on a narrower age-range or directly compare several defined periods to directly examine changes in infant-adult interbrain synchrony in the alpha band across the first year of life.

An additional important aspect that should be noted is that paternal body odor was compared to a blank shirt, thus it is possible that the effects are not specific to paternal body odor, but could reflect the presence of human odor in general. That being said, infants were continuously exposed to other human odors, including those of the stranger interacting with them, and due to the ecological nature of the paradigm the experimental environment was far from sterile, which further supports the observed effects.

Moreover, the effects of paternal body odor may operate through several mechanisms. Paternal odors may exert subliminal influences on infants’ behavior and neural responses without conscious awareness, and it may also be consciously perceived and recognized by infants due to familiarity with their fathers’ scent. Another important aspect not directly examined in the current study is the possibility that emotional states, prior to falling asleep and during the body-odor collection period may alter the chemical composition of the odor and, in turn, influence infants’ responses. To address this, future studies should assess the emotional state of body-odor donors during the collection period (e.g., via cortisol levels and self-reported anxiety or mood questionnaires) and examine how infants’ responses vary accordingly.

In addition, given the possibility of olfactory habituation during the continuous exposure to paternal BO, we examined whether interbrain synchrony and arousal differed between the first and second minutes of the interaction. We found no differences in either neural or behavioral measures between these time windows (see fig. S5). While the direct impact of olfactory cues may be strongest in the initial seconds following exposure, it is possible that their influence on the subsequent interaction with the stranger, including neural synchrony and behavioral measures, persists over time, even as some degree of habituation may occur. We acknowledge that more fine-grained analytical approaches may be required in future work to capture dynamic changes in interbrain synchrony related to exposure to olfactory cues.

It is important to note that our study represents the first effort to test paternal body odors effects on neural synchrony and future research should compare odors stemming from a variety of sources, including other family members, and should systematically vary odor intensity and pleasantness. Paternal body odor likely represents one component of a broader ensemble of social cues that together influence infant-adult neural synchrony and behavioral responses.

In sum, our study shows that infants are sensitive to their father’s odor and respond to his olfactory cues by increasing brain-to-brain synchrony with a male member of the social group. As such, our results highlight important processes by which a father’s sensory cues contribute to his infant’s brain maturation. Father’s odors invoke special interbrain rhythms and a distinct interbrain connectivity pattern that push the infant’s development forward, consolidate a later-emerging brain rhythm, support attention regulatory processes, and open the child to more stimulating and exploration-based social interactions. Our results indicate that olfactory associative learning is not limited to maternal odors or to the in-utero exposure to maternal chemosignals, and father odors can trigger associative learning that link paternal odors with positive arousal. Much further research is needed to understand how fathers impact their infants’ brain development, how children store their father’s sensory cues and utilize them to grow a social brain, and what are the neural and chemical pathways by which the scent of a father is retained in its absence to form memories, build attachments, and help children grow, explore, and expand their cognitive and social world.

## MATERIALS AND METHODS

The study was preregistered: https://osf.io/dhcx4/overview?view_only=a6d87837baac47a1b1968308739d2ca5.

Please note several differences between the current study and the preregistration. While the main hypotheses and general analytic approach followed the preregistered plan, several deviations are reported for transparency in the Supplementary Text.

### Participants

All procedures used in this study including paradigms, questionnaires, and equipment were approved by the Reichman University IRB committee (P_2022053) and all fathers signed informed consent. All procedures were explained to the fathers before the study and were performed in accordance with ethical guidelines. Participants were free to leave the experiment at any time with full compensation. Participants were recruited through online forums and social media groups. Inclusion criteria were: father above 18 years of age, infant is the father’s biological child, both father and infant are healthy at the time of the experiment and are not diagnosed with epilepsy. Overall, 110 participants [55 fathers and 55 infants, fathers’ age, M = 34.86 years, SD = 5.52 years infants’ age, M = 7.93 months, SD = 1.73 months, range = 5.0–12.6 months; infants’ gender: 23 female (F) and 32 male (M)] arrived to the lab. Yet, different numbers of participants completed each paradigm. Two participants were excluded due to technical issues. In the current study of 47 participants that completed all three experimental conditions, seven were excluded; Five participants were excluded because the baby cried for most of the interaction during one of the conditions, resulting in the condition being stopped. Additionally, one participant was excluded due to a low number of final epochs in one of the conditions after preprocessing (fewer than 50 epochs). Finally, one participant was excluded after the calculation of connectivity scores, as their average connectivity score was more than 3 standard deviations above the group mean, resulting in 80 participants (40 dyads) included in the current analysis.

A power analysis was conducted using G*Power software based on effect sizes reported in our previous within-subject study comparing interbrain synchrony between body odor and blank conditions. Assuming a medium effect size (d ≈ 0.5), a significance level of α = 0.05, and 80% power, the required sample size is estimated to be at least 34 participants.

### Procedure

#### Body odor collection

Fathers were provided with 100% cotton T-shirts. The donors were instructed to wear the shirt for two consecutive nights before the experiment day. Fathers were instructed to refrain from consuming foods known to affect body odor for at least two days prior to wearing the shirt. Fathers were further asked not to use soap, shampoo, conditioner, or deodorant before they wear the shirt. Between the two nights, the T-shirts were kept inside closed glass jars that were stored in the fathers’ home freezer. On the second night, fathers wore the same shirt again, without showering between the two nights.

#### Paradigm

Both the Infant and the adult (father/male experimenter) were fitted with EEG electrodes. The male experimenter (stranger) was not present in the room before the actual interactions with the infants began, in order to avoid any prior familiarization with the infant.

During the experiment the infants were sitting in a high baby chair or in a baby bouncer chair, depending on their ability to sit by themselves. The T-shirts (Body Odor/Blank) were placed under the infants’ neck in the cases they were sitting in the baby bouncer chair, or on the feeding tray of the high chair. All paradigms were videotaped for later offline coding. The experiment included the following paradigms:

Father-infant free interaction Paradigm: Infant-father face-to-face free interaction. Infant and father were left alone in the experiment room. Fathers were instructed to interact with the infant as they normally do, avoiding physical contact or using objects such as toys or pacifiers. Interactions were filmed for 3 min.

Stranger-infant free interaction Paradigm: Infant-stranger face-to face free interaction. An infant and an unfamiliar male (the stranger) were left alone in the experiment room. Two sequential interactions were conducted. In both the stranger was instructed to have a face-to-face free interaction with the infant, without physical contact or use of toys. At the beginning of each interaction, the stranger father opened one of two glass jars consisting of T-shirts either with the infant’s father Body Odor (BO) or a blank new T-shirt. The two odor conditions were counterbalanced. The T-shirts (Body Odor/Blank) were placed under the infants’ neck in case they were sitting in the baby bouncer chair, or on the feeding tray of the highchair. The duration of each interaction was approximately 3 min.

### Randomization and blinding

Odor conditions were counterbalanced in order across participants. The experiment was double-blind, i.e., all participants (infants, stranger and fathers) were blind to experimental conditions.

### Dual-EEG data acquisition

Neuroelectric activity in both participants of each dyad was simultaneously and continuously recorded, using Brain Products GmbH. The system was composed of two Acticap helmets with 16 active electrodes arranged according to the international 10/20 system. The reference is fixed on FCz. The impedances were maintained below 10 kΩ. Both subjects were connected to the same amplifier that guaranteed millisecond-range synchrony between the two EEG recordings.

### EEG artifacts and analysis

Preprocessing was conducted using Python in Anaconda with the MNE software suite (0.24.1). First, for the preprocessing procedure, we separated the EEG data file of each dyad to infant data file and adult data file. Then, we applied a 1- to 50-Hz band-pass filter. Next, following segmentation of the signal to 1-s epochs with 500-ms overlap between epochs, we applied an automatic algorithm that detects noisy segments. Similar to previous hyperscaning EEG research (*24*, *73*, *74*) we used the MNE “AutoReject” algorithm (*75*). The AutoReject algorithm removes trials containing transient jumps in isolated channels but does not necessarily work well for a systematic physiological artifact that affects multiple sensors. For these purposes, we used MNE’s implementations of FastICA and CORRMAP (*76*). CORRMAP allows manual selection of an independent component (IC) for exclusion in one participant and use the chosen component as a template for selecting and excluding similar components in other participants.

### Connectivity analysis

To compare the intersubject connectivity scores between two conditions (BO versus blank, and father-infant versus stranger (blank), the duration of each dyad in both conditions was matched by taking the minimal duration of both conditions for each dyad. Following EEG preprocessing, the epochs of both subjects (adult and infant) in each paradigm were matched such that only data points with “good” epochs for both subjects were included.

Consequently, if a participant did not complete the full duration included in the calculations for one condition but did complete it in the other, the connectivity estimates for the Stranger-Infant blank condition may vary slightly, as they are computed from a different number of epochs in the comparisons to BO and to father-infant.

For the 40 dyads included in the final analysis, there was no difference between conditions in number of final epochs used for connectivity computation [Father-infant: 173.2 ± 41.4, Stranger-infant: 187.13 ± 33.5, *t*(39) = 1.6, *P* = 0.11; BO: 191.7 ± 38.9, blank: 186.3 ± 33.5, *t*(39) = 0.8, *P* = 0.43].

The adult-infant interbrain neural connectivity values were calculated for theta (4 to 7 Hz) and alpha (8 to 12 Hz) frequency bands. Consistent with recent hyperscanning studies (*23*, *24*, *74*), we wished to avoid spurious hyper-connections that could result from similarity in the sensory experiences of the participants and that are not related to the social interaction itself. We therefore decided to use the weighted phase lag index (wPLI) as our measure of inter-brain synchrony, a measure that is an extension of the PLI. By weighing each phase difference according to the magnitude of the lag, phase differences around zero only marginally contribute to the calculation of the wPLI. This procedure reduces the probability of detecting “false positive” connectivity in the case of noise sources with near-zero phase lag and increases the sensitivity in detecting phase synchronization (*77*). wPLI ranges between 0 and 1, where 0 indicates no synchrony and 1 full synchrony.

Based on our previous findings (*24*) and to reduce multiple comparisons, we choose to focus on interbrain synchrony in infants’ temporal regions and adults’ central and temporal regions; Right Temporal (RT- T8, P8), Left Temporal (LT- T7, P7), Right Central (RC - C4) and Left Central (LC- C3) regions. These regions were previously indicated to be involved in interbrain neural synchrony, particularly in studies using more ecological settings. Overall, this resulted in 8 possible combinations of adult-infant interbrain links.

### Statistics

We used a conservative approach, applying nonparametric Wilcoxon signed-rank tests to all eight possible infant-adult connectivity combinations to evaluate which interbrain links facilitated the differences in interbrain synchrony between the two experimental conditions. All results were Bonferroni-corrected to account for the eight comparisons.

### Social interaction behavior analysis

Coding was conducted offline by coders trained to reliability who were blind to all other information, including the odor condition. For the behavioral analysis we included only subjects with codes in the two stranger conditions. Due to technical issues, coding was completed for 35 participants in each behavioral coding methods. We used two behavioral coding methods:

1) Micro-level second-by-second. Coding scheme previously validated in our lab and consistent with prior research that showed correlations between these micro-level behaviors and brain activations. Here, we explored the effects of fathers’ BO on *infant social arousal*. Social arousal was defined as the relative proportion of time in which at least one of the following behaviors occurred: changes in body posture indicating increased orientation to social interactions (leaning forward or backward rather than sitting upright), high positive affect (smiling or laughing), vocalizations (cooing), or active exploration of the father’s shirt (holding or playing, see fig. S7 for direct examination of infant interaction with the father’s shirt). Consistent with previous research, we measured *infant visual attention*. For the visual attention measurement, we calculated the exact proportion duration that infants gazed toward the adult, including moments of joint attention and excluding moments of crying and fussing.

2) The Coding Interactive Behavior (CIB). The CIB is a global rating system of social interaction comprises of multiple scales scored from 1 (low) to 5 (high) that combine into theoretically-derived constructs. The CIB has been validated in a large number of studies in 33 countries of healthy and high-risk populations. The system has shown construct validity, test-retest reliability, and prediction to multiple social, cognitive, biological and neural outcomes. The CIB utilizes scales for parent, for infant, and for the dyadic atmosphere. In the current study we analyzed Infants’ safety and engagement. Infants Safety and Engagement score was the average of the following parameters: adult supportive presence, infant positive affect, infant initiation of social interactions, infant-lead interaction, dyadic reciprocity, dyadic adaptation-regulation, and a relaxed dyadic atmosphere.

Correlations among behavioral measures are reported in fig. S8.

An exploratory analysis was conducted to examine potential correlations between behavioral measures and interbrain neural synchrony. For each participant, a change score was calculated for both types of measurements: the difference between odor conditions divided by the maximum value of both conditions: $\frac{\mathrm{BO}-\text{BLANK}}{\text{MAX}\left(\mathrm{BO},\text{BLANK}\right)}$. This measure captures within-dyad differences between conditions while accounting for inter-dyad differences in overall synchrony magnitude.

### Paternal involvement in childcare

Paternal involvement was assessed using a questionnaire the fathers completed at the day of the experiment. Of the 40 father-infant dyads who were included in the final analysis, 34 fathers completed a questionnaire assessing their involvement in childcare at home. Fathers were asked to estimate their relative involvement in childcare compared to the other parent (proportion) and to rate the extent to which they care for the child on a 1–5 scale. To derive a paternal involvement score, all questionnaire responses were z-scored, and the two measures for each father were then averaged to obtain a mean involvement score. An exploratory analysis was conducted to examine whether paternal involvement was associated with the effect of paternal BO on interbrain synchrony.
