# Supplementary material for: Scent of a father: Paternal body odors boost interbrain synchrony
Source: Sci Adv. 2026 Jul 15;12(29):eaed6110. doi: 10.1126/sciadv.aed6110 (PMC13371932; doi:10.1126/sciadv.aed6110)
Supplement: Supplementary file 1 — Supplementary Text Figs. S1 to S8 [file sciadv.aed6110_sm.pdf]

Science Advances
AAAS

Supplementary Materials for

Scent of a father: Paternal body odors boost interbrain synchrony

Yaara Endevelt-Shapira *et al.*

Corresponding author: Yaara Endevelt-Shapira, yaara.yaara@gmail.com

Sci. Adv. **12**, eaed6110 (2026)
DOI: 10.1126/sciadv.aed6110

This PDF file includes:

Supplementary Text
Figs. S1 to S8

Supplementary Text

<u>Differences between the current study and the preregistration</u>

In the preregistration, we hypothesized that interbrain synchrony would not be restricted to right–right hemispheric connections, as reported in our previous mother–infant hyperscanning study. Accordingly, in the current study we examined all possible combinations of cross-hemispheric and intra-hemispheric interbrain connections. Although we did not conduct a direct statistical comparison between cross- and intra-hemispheric connections, all interbrain connections were tested across experimental conditions, including both inter-and intra-hemispheric connections and the observed patterns were interpreted in relation to prior findings.

In addition, although the preregistration explicitly stated that "we intend to calculate connectivity in the theta and alpha frequency bands," it did not specify hypotheses regarding differences between alpha- and theta-band synchrony. In the current study, we included an additional hypothesis examining potential differences between alpha- and theta-band interbrain synchrony.

Importantly, we attempted to complete the back-to-back condition as specified in the preregistration; however, many infants did not complete the baseline paradigm due to infant distress (e.g., crying or fussiness), limited engagement with the screen (e.g., searching for the father), or the need for soothing (e.g., pacifier use, holding toys, or eating). In some cases, sessions were interrupted because infants pulled at EEG electrodes, or exhibited excessive movement, resulting in poor signal quality. Additional interruptions occurred when fathers attempted to calm or redirect the infant or spoke to the infant during the paradigm, thereby compromising the validity of the baseline condition.

Finally, Infant behavior toward the T-shirt was coded using a microcoding scale, with second-by-second coding of moments when the infant held or played with the T-shirt, rather than the CIB global coding specified in the preregistration. Active exploration of the father's shirt (holding, playing) was included as one of the variables in the infant Social Arousal measure.

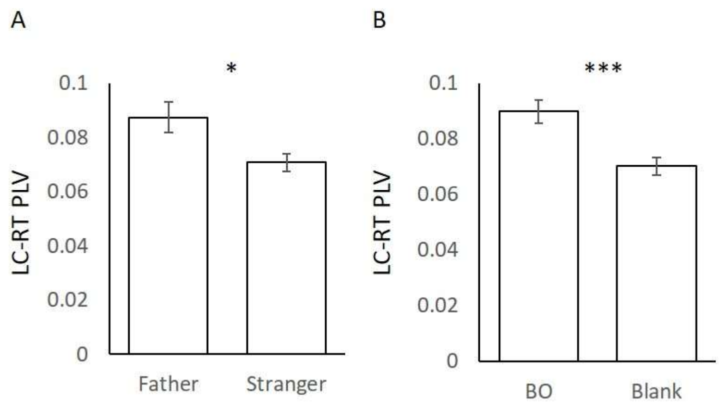

Fig. S1. Phase-locking value (PLV) analyses replicated the wPLI findings in both the father–stranger and BO–blank comparisons.

To test the robustness of the effect with an alternative connectivity measure, we repeated the analysis using the PLV method. The analysis in alpha frequency band (8-12 Hz) revealed (**A**) significantly higher father-infant interbrain connectivity compared to the stranger-infant interaction. (N = 40, Father-infant: 0.087 ± 0.035, Stranger-infant: 0.071 ± 0.020, Z = 2.31, W = 582, $P = 0.02$), and (**B**) significantly higher connectivity in BO condition relative to blank between the left central area of the stranger and the right temporal area of the infant (N = 40, BO: 0.090 ± 0.026, blank: 0.070 ± 0.020, Z = 3.3, W = 658, $P = 0.0006$).

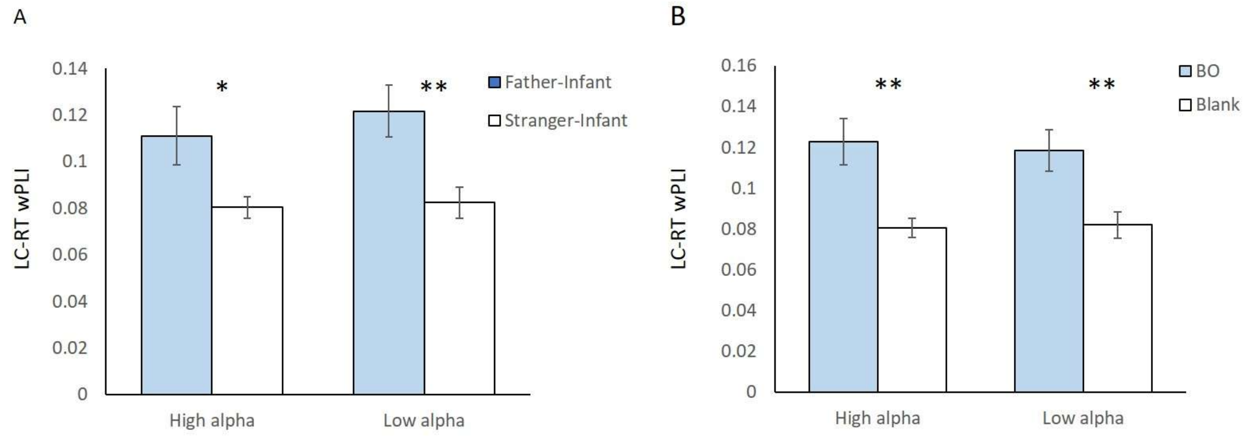

Fig. S2. Interbrain synchrony in low and high alpha frequency bands

To test the robustness of the effect across frequency boundaries, wPLI scores were calculated separately for the low (8–10 Hz) and high (10–12 Hz) alpha bands. The analysis revealed significantly higher interbrain connectivity in the (**A**) father–infant interbrain compared to the stranger–infant connectivity in both the high alpha band (N = 40, Father-infant: 0.11 ± 0.08, stranger: 0.08 ± 0.03, Z = 2.3, W = 579, *P* = 0.02) and the low alpha band (N = 40, Father-infant: 0.12 ± 0.07, stranger: 0.08 ± 0.04, Z = 3.0, W = 635, *P* = 0.002). Similarly,(**B**) in the BO condition the connectivity was significantly higher than Blank condition between the stranger's left central region and the infant's right temporal region in both the high alpha (N = 40, BO: 0.12 ± 0.07, blank: 0.08 ± 0.03, Z = 3.03, W = 636, *P* = 0.002) and the low alpha (N = 40, BO: 0.12 ± 0.06, blank: 0.08 ± 0.04, Z = 2.9, W = 623, *P* = 0.004) frequency bands.

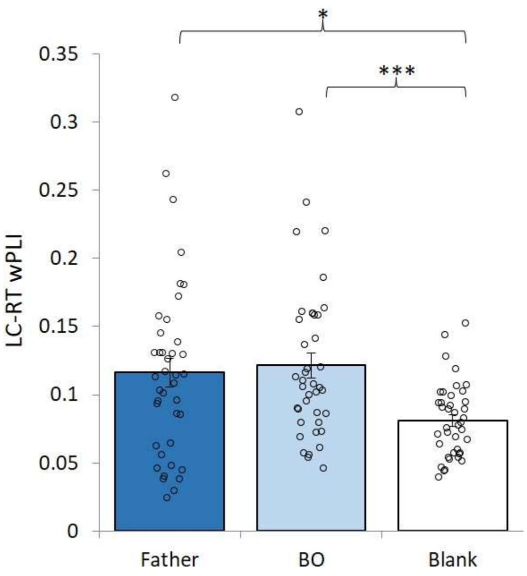

Fig. S3. Interbrain synchrony in the paternal BO condition is comparable to the father–infant condition. Interbrain neural synchrony scores between the left central area of the adult and the right temporal area of the infant were calculated for the three conditions (father-infant, stranger-infant with paternal body odor (BO), and stranger-infant in blank condition (Blank)), using the same minimum duration for each dyad across the three conditions. Comparison between the three conditions revealed significantly higher father-infant interbrain connectivity compared to the stranger-infant (Blank) interaction (Z = 2.8, W = 616, $P = 0.005$, *P corrected* = 0.015), and significantly higher connectivity in BO condition relative to blank condition (Z = 3.98, W = 706, $P < 0.001$, *P corrected* < 0.001). No significant difference was observed between father-infant and BO conditions in neural synchrony scores (Z = 0.44, W = 377, $P = 0.67$). *$P < 0.05$, ***$P<0.001$.

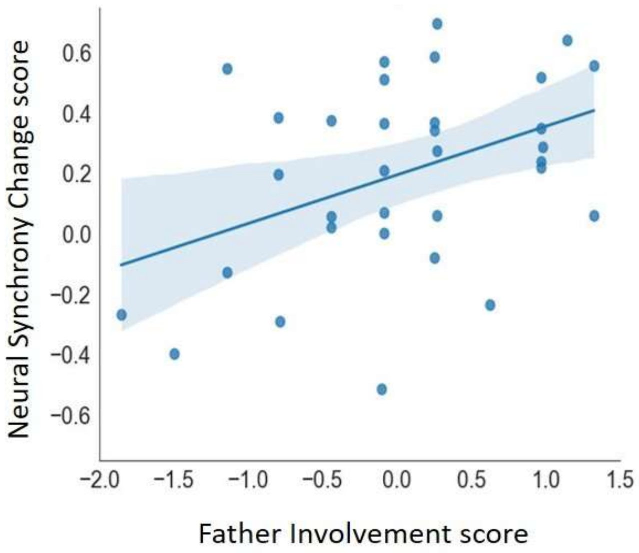

Fig. S4. Paternal involvement in childcare is associated with the effects of BO on interbrain synchrony. To examine how individual differences influence paternal odor effects, we assessed father involvement using a questionnaire the fathers completed at the day of the experiment.
Of the 40 father–infant dyads who were included in the final analysis, 34 fathers completed a questionnaire assessing their involvement in childcare at home. Fathers were asked to estimate their relative involvement in childcare compared to the other parent and to rate the extent to which they care for the child on a 1–5 scale.
To derive a paternal involvement score, all questionnaire responses were z-scored, and the two measures for each father were then averaged to obtain a mean involvement score. To examine whether paternal involvement was associated with the effect of paternal BO on interbrain synchrony, a Spearman correlation analysis was conducted. One outlier was excluded from the analysis. The results revealed a significant correlation between paternal involvement scores and interbrain synchrony change scores (N = 33, $r_s = 0.38$, $P = 0.03$).

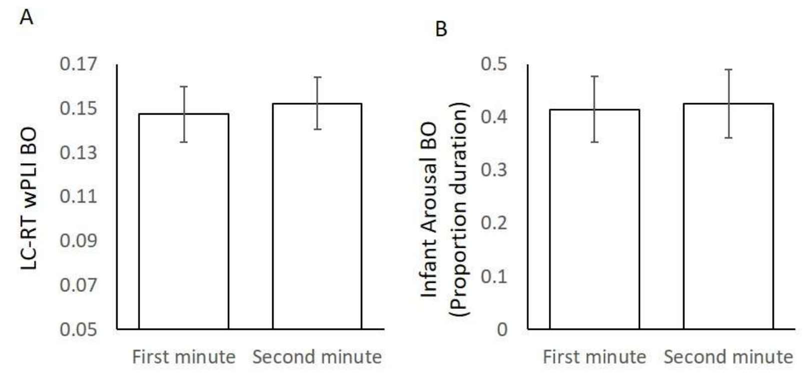

Fig. S5. No difference in interbrain synchrony and arousal between the first and the second minute of the BO condition.

To examine whether interbrain synchrony and infants' arousal in the BO condition differed over time, we (A) computed wPLI scores separately for the first and second minutes of the interaction and compared interbrain synchrony between these two time windows. This analysis revealed no significant difference between the first and second minutes ($W = 397$, $Z = 0.18$, $P = 0.87$).
(B) computed the proportion of positive arousal for the first and the second minute of the interaction, and compared between these two time windows. This analysis also revealed no significant difference between the first and second minutes ($W = 226$, $Z = 0.54$, $P = 0.60$).

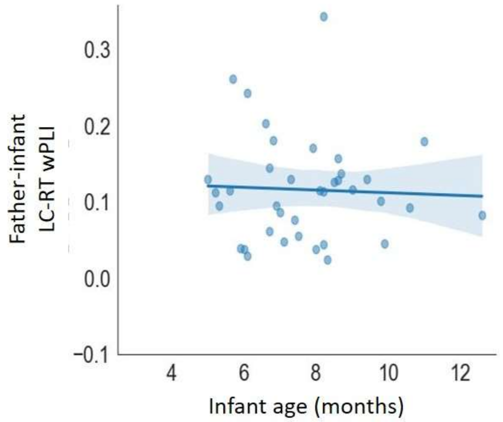

Fig. S6. Infant age is not associated with infant-father interbrain synchrony scores.
To examine whether infant age is associated with the level of infant-father interbrain synchrony, a Spearman correlation analysis was conducted. The analysis revealed no significant correlation between infant age and infant-father interbrain synchrony scores ($r_s = 0.008$, $P = 0.96$).

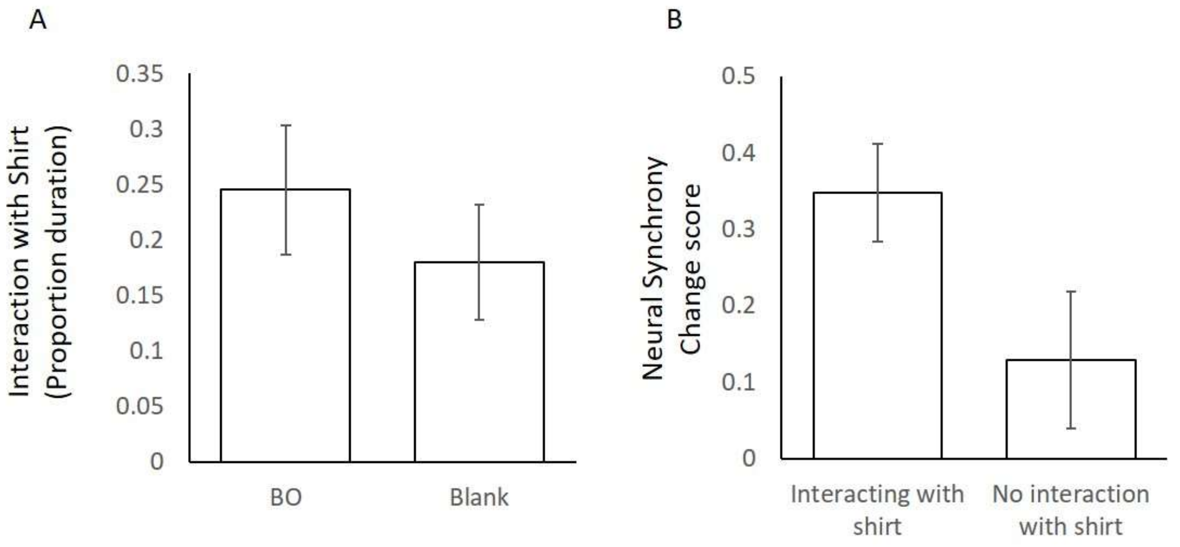

Fig. S7. Infants' interaction with the shirt.

To explore whether infants' interaction with the t-shirt was different between BO and Blank conditions, moments of holding or playing with the t-shirt were coded. (**A**) The analysis revealed no significant difference between conditions (N = 35, BO = 0.25 ± 0.34, Blank = 0.18 ± 0.30, W = 117, Z = 1.5, $P = 0.14$). (**B**) Dyads were divided into two subgroups based on whether infants interacted with the shirt during the BO condition, defined as a proportion of interaction duration greater than 0.01, versus no interaction (≤ 0.01), resulting in groups of 22 and 13 dyads, respectively. Inter-brain synchrony change scores were then compared between the two subgroups using a Mann–Whitney U test. This analysis revealed a trend toward higher neural synchrony in the group that interacted with the shirt (no shirt interaction: 0.13 ± 0.31; shirt interaction: 0.35 ± 0.29; W = 90, $P = 0.07$).

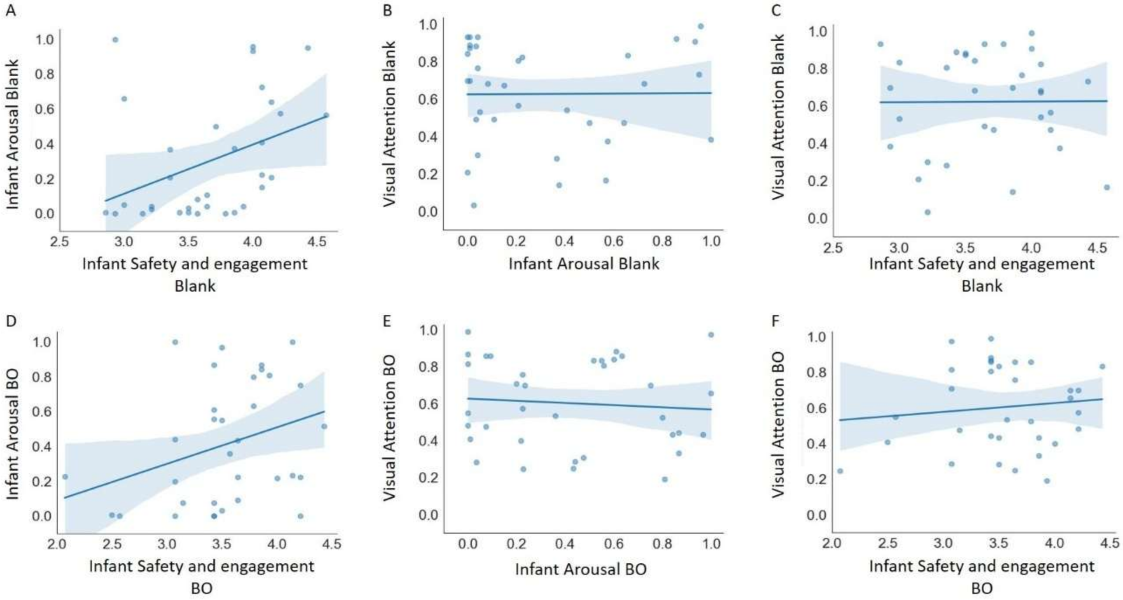

Fig. S8. Correlations among behavioral measures

To examine potential correlations among the three different infant behavioral measures, Spearman rank correlations were used. In the Blank condition, (**A**) infant arousal and infant safety were significantly correlated ($r_s = 0.47$, $P = 0.006$). However, infant visual attention was not correlated with either (**B**) infant arousal ($r_s = -0.12$, $P = 0.51$) or (**C**) infant safety ($r_s = -0.05$, $P = 0.79$). In the BO condition, (**D**) infant arousal and infant safety showed a trend toward a correlation ($r_s = 0.31$, $P = 0.08$). However, infant visual attention was not correlated with either (**E**) infant arousal ($r_s = -0.11$, $P = 0.55$) or (**F**) infant safety ($r_s = -0.06$, $P = 0.72$).
